# The Electrophysiological Correlates of Phoneme Perception in Primary Progressive Aphasia: A Preliminary Case Series

**DOI:** 10.3389/fnhum.2021.618549

**Published:** 2021-06-02

**Authors:** Jara Stalpaert, Marijke Miatton, Anne Sieben, Tim Van Langenhove, Pieter van Mierlo, Miet De Letter

**Affiliations:** ^1^Department of Rehabilitation Sciences, Faculty of Medicine and Health Sciences, Ghent University, Ghent, Belgium; ^2^Department of Neurology, Ghent University Hospital, Ghent, Belgium; ^3^Department of Electronics and Information Systems, Medical Image and Signal Processing Group, Ghent University, Ghent, Belgium

**Keywords:** primary progressive aphasia, electrophysiology, event-related potentials, MMN, P300, phoneme perception, diagnosis

## Abstract

**Aims**: This study aimed to investigate phoneme perception in patients with primary progressive aphasia (PPA) by using the event-related potential (ERP) technique. These ERP components might contribute to the diagnostic process of PPA and its clinical variants (NFV: nonfluent variant, SV: semantic variant, LV: logopenic variant) and reveal insights about phoneme perception processes in these patients.

**Method**: Phoneme discrimination and categorization processes were investigated by the mismatch negativity (MMN) and P300 in eight persons with early- and late-stage PPA (3 NFV, 2 LV, 2 SV, and 1 PPA-NOS; not otherwise specified) and 30 age-matched healthy adults. The mean amplitude, the onset latency, and the topographic distribution of both components in each patient were compared to the results of the control group.

**Results**: The MMN was absent or the onset latency of the MMN was delayed in the patients with the NFV, LV, and PPA-NOS in comparison to the control group. In contrast, no differences in mean amplitudes and onset latencies of the MMN were found between the patients with the SV and the control group. Concerning the P300, variable results were found in the patients with the NFV, SV, and PPA-NOS, but the P300 of both patients with the LV was delayed and prolonged with increased mean amplitude in comparison to the control group.

**Conclusion**: In this preliminary study, phoneme discrimination deficits were found in the patients with the NFV and LV, and variable deficits in phoneme categorization processes were found in all patients with PPA. In clinical practice, the MMN might be valuable to differentiate the SV from the NFV and the LV and the P300 to differentiate the LV from the NFV and the SV. Further research in larger and independent patient groups is required to investigate the applicability of these components in the diagnostic process and to determine the nature of these speech perception deficits in the clinical variants of PPA.

## Introduction

Primary progressive aphasia (PPA) refers to a heterogeneous group of neurodegenerative clinical syndromes in which a range of language and speech abilities progressively deteriorate with relative preservation of the other cognitive domains. An international group of researchers provided a framework for the diagnosis of PPA and its clinical variants (Gorno-Tempini et al., 2011). In this framework, diagnostic criteria for three clinical variants, namely the nonfluent or agrammatic variant (NFV), the semantic variant (SV), and the logopenic variant (LV), are provided. The NFV is characterized by the presence of agrammatism in language production, apraxia of speech (AOS), impaired comprehension of syntactically complex sentences, and spared single-word comprehension and object knowledge. The key characteristics of the SV are impaired confrontation naming and single-word comprehension. Furthermore, an impaired object knowledge, surface dyslexia or dysgraphia, a spared repetition (but regularization errors may occur), and a spared speech production could be present. The language pattern of the LV typically shows impaired single-word retrieval and impaired repetition of sentences and phrases. In this variant, phonological errors could be present but the single-word comprehension, object knowledge, and motor speech production are mostly spared. The clinical diagnosis of PPA can be imaging-supported or supported by a definite pathology. Although the clinicopathological correspondence of PPA is not absolute, the two most common neuropathological correlates of PPA are Alzheimer’s disease (AD) and frontotemporal lobar degeneration (FTLD). The SV and NFV are most frequently associated with FTLD pathology and the LV with AD pathology, but other pathologies have been found as well (Davies et al., 2005; Kertesz et al., 2005; Amici et al., 2006; Grossman, 2010; Leyton and Ballard, 2016).

Despite the availability of this diagnostic framework, the clinical and pathological overlap between PPA and other neurodegenerative diseases complicates the diagnostic process (Leyton and Ballard, 2016; Johnen et al., 2018). Furthermore, some patients with the root diagnosis of PPA cannot be classified into these three clinical variants due to too mild, too severe, or mixed deficits from the early to the later disease stages. In particular, the differentiation between the NFV and the LV remains complicated in the early and later disease stages due to overlapping language and speech characteristics (Leyton and Hodges, 2014; Botha and Josephs, 2019; Utianski et al., 2019; Stalpaert et al., 2020). This clinical differentiation between variants is important because these variants may function as clinical markers suggesting the most likely pathology (Grossman, 2014). Consequently, an accurate and timely clinical and/or etiological diagnosis may support patients and their social support system in planning speech-language therapy, mobilizing the appropriate social services, and anticipating specific problems which might co-occur in the future. In addition, this diagnosis offers the opportunity to investigate disease-modifying treatments since currently, no pharmacological treatments are available (Grossman, 2014; Leyton and Ballard, 2016; Vandenberghe, 2016; Henry and Grasso, 2018; Marshall et al., 2018).

In the three variants, language comprehension deficits are reported, although with disturbances in various underlying processes (Gorno-Tempini et al., 2011; Harciarek et al., 2014; Leyton and Hodges, 2014). A correct phoneme perception is a prerequisite and an important aspect of language comprehension. Phonemes are the smallest units of spoken language characterized by specific combinations of spectrotemporal auditory features (Hardy et al., 2017b). The term “phoneme perception” refers to a set of perceptual, acoustic, and linguistic processes to detect, discriminate, categorize, and identify phonemes. Since the analysis of phonemes is one of the first processing steps in language comprehension, deficits may lead to difficulties in the consecutive language processes (Aerts et al., 2015).

Studies investigating phoneme perception and more generally speech perception in patients with PPA are very limited and they all investigate various aspects of this process by behavioral assessments, functional magnetic resonance imaging (fMRI), electroencephalography (EEG), and magnetoencephalography (MEG). Johnson et al. (2020) evaluated phoneme discrimination behaviorally in the three variants of PPA. Based on the results of this study, the authors suggested that phoneme discrimination assessments may help differentiate the LV from the NFV and SV. Alterations in the detection of phonemic spectral structures were also found in patients with the LV by an fMRI experiment. However, deficits in phonemic spectral structure processes were also found in patients with the NFV and the SV (Hardy et al., 2017a,b). Furthermore, the processing of speech signal temporal regularity was only disturbed in the patients with the NFV, and the analysis of prosodic predictability, an index of the fundamental, non-linguistic information content of speech signals, was impaired in patients with the SV and NFV (Hardy et al., 2017a,b). Cope et al. (2017) investigated speech perception in patients with the NFV by EEG and MEG. They addressed speech perception in the context of the predictive coding framework. According to this framework, speech perception relies on the integration of sensory signals and prior knowledge and expectations. Predictions of expected sensory information are expressed in top-down connections, while prediction errors are passed forward by bottom-up processes to update the model. The authors found that the reconciliation of predictions in the temporal cortex was delayed in patients with the NFV which results in an inflexible application of prior expectations. Furthermore, the predictions were excessively precise which may explain the agrammatism and subjective difficulties with speech perception.

Besides phoneme and speech perception processes, nonverbal sound processes were also investigated in patients with the three variants of PPA by several studies. In the study of Hardy et al. (2019), patients with the NFV performed worse on pure-tone audiometry than healthy participants and patients with AD, and an increased interaural functional asymmetry was found in the patients with the NFV. These results suggest the presence of both peripheral and more central auditory deficits in patients with NFV. In other studies, early perceptual auditory deficits such as an impaired temporal analysis were also most frequently found in the NFV, while the nonverbal sound processing deficits in the SV were most frequently auditory associative deficits and deficits in sound meaning. In the LV, one study proposed that the auditory deficits might be influenced by nonverbal memory function and another did not find significant deficits (Bozeat et al., 2000; Goll et al., 2010, 2011; Rohrer et al., 2012; Golden et al., 2015; Grube et al., 2016; Hardy et al., 2017a).

Based on the above-standing results, no general conclusions about the phoneme perception deficits in the variants of PPA can be drawn. However, it might be important to gain insight into these processes and their influence on the consecutive language processes to reveal the underlying processing deficiencies in language comprehension. Furthermore, differences in phoneme perception processes might help to differentiate between variants in the diagnostic process of PPA. Phoneme perception assessments are often not included in the standard language evaluations administered in patients with PPA. Consequently, rehabilitating speech perception deficits is not the focus of speech and language therapy, complaints of speech perception deficits might be falsely attributed to hearing loss, and the patients might be offered inappropriate hearing amplification interventions (Johnson et al., 2020).

An objective, non-invasive method to investigate phoneme perception processes is the event-related potential (ERP) technique. ERPs represent small voltage fluctuations in the continuous EEG that are time-locked to a particular event (Luck, 2005). Two auditory ERP components that can be associated with phoneme perception are the mismatch negativity (MMN) and the P300. The MMN and the P300 are elicited respectively by a pre-attentive and attentive auditory oddball paradigm in which a deviant stimulus infrequently occurs in a sequence of standard stimuli. The most frequently used stimuli to elicit these components are tones, but phonemes can be used as well (Aaltonen et al., 1987; Näätänen et al., 1997; Dehaene-Lambertz and Baillet, 1998; Aerts et al., 2013). The MMN is a negative response to the deviant stimuli that typically peaks between 160 and 220 ms with a frontocentral topographic distribution. The most widely accepted theory is that this component indexes auditory discrimination processes in which the deviant stimulus is compared to a short–term memory trace of the standard stimuli (Luck, 2005; Alain and Tremblay, 2007; Picton, 2011; Luck and Kappenman, 2012; Näätänen et al., 2012). Concerning phonemes, MMN data indicate that phoneme perception is based on language-specific phoneme traces which represent the acoustic properties of the various phonemes of the native language (Näätänen, 2001; Becker and Reinvang, 2007). The P300 (or P3) component is a positive response that occurs at 300–600 ms after the presentation of the deviant stimulus with a parietal topographic scalp distribution. This component is associated with auditory stimulus categorization and components of attention and working memory (Kok, 2001; Bledowski et al., 2004; Luck and Kappenman, 2012). In terms of phoneme perception, the MMN can be associated with phoneme discrimination and the P300 can be associated with phoneme categorization.

Phoneme discrimination and categorization have not yet been investigated in patients with PPA by the MMN and P300 components (Stalpaert et al., 2020). Differences in latency, amplitude, and topographic distribution of the MMN and P300 components might reveal information about possible deficits in the phoneme perception processes. Consequently, this might help to gain insight into the nature of the language comprehension deficits in patients with the three clinical variants of PPA. Furthermore, these ERP components might contribute to the diagnostic process of PPA and its clinical variants. Therefore, this study aimed to investigate speech perception processes by the MMN and P300 components in patients with the three variants of PPA.

## Materials and Methods

### Participants

Eight patients with a clinical diagnosis of PPA (five male and three female) were recruited at the Department of Neurology of the Ghent University Hospital. The clinical diagnosis of PPA was made by experienced neurologists (TL and AS) and speech-language pathologists (MDL and JS) based on the case history, the neurological evaluation, the neuroimaging results, and the evaluation of the language and speech abilities. All patients met the criteria for the root diagnosis of PPA and were classified into the clinical variants following the criteria of Gorno-Tempini et al. (2011). For this study, the language and speech abilities of the patients were evaluated by spontaneous speech, the Dutch version of the Comprehensive Aphasia Test (CAT-NL; Swinburn et al., 2014), and the Diagnostic Instrument for Apraxia of Speech (DIAS; Feiken and Jonkers, 2012). Furthermore, the Montreal Cognitive Assessment (MoCA; Nasreddine et al., 2005) and the Dutch Handedness Inventory (DHI; Van Strien, 1992) were administered to each included patient. The included cases were divided into early- (≥23) and late-stage (<23) PPA based on their result on the MoCA (Carson et al., 2018). The peripheral hearing function of each patient was screened by pure-tone audiometry and tympanometry. The tympanometry of all included patients with PPA was within the normal range. The results of the pure-tone audiometry showed a sensorineural age-related hearing impairment in each patient, indicated by increased high-frequency auditory thresholds. The pure tone average (PTA; average air conduction hearing thresholds at 500, 1,000, 2,000, and 4,000 Hz) of the best ear of each patient is presented in the Results section of this article.

The age-matched healthy control (HC) group consisted of 30 right-handed adults recruited by snowball sampling (five male and five female participants per age decade: 50–59 years, 60–69 years, 70–79 years). The age of the HCs ranged from 50–78 years with a mean of 63.9 years (SD = 8.31) and the education level ranged from 10–17 years with a mean of 14 years (SD = 2.05). The MoCA and the DHI were performed in all HCs. The scores for the MoCA ranged from 26 to 30 with a mean score of 28.4 (SD = 1) and all HCs were right-handed (score of +10 on the Dutch Handedness Inventory). Furthermore, the HCs had a (corrected-to-)normal vision and reported no subjective complaints of hearing loss. None of the HCs had a history of neurological disorders, developmental learning or language disorders, and psychiatric disorders. The native language of all participants was Dutch. The study was performed in accordance with the Declaration of Helsinki and approved by the Ethics Committee of the University Hospital Ghent. Informed consent was obtained from all the participants.

### Experimental Procedure

An inattentive and attentive auditory oddball paradigm were administered in the patients with PPA and the HC group in a dimly illuminated room in the Ghent University Hospital. The EEG was recorded for each participant in the morning or early afternoon. The auditory stimuli and oddball paradigms were adapted from Aerts et al. (2013). The standard stimulus [bǝ] differed from the deviant stimulus [gǝ] by one phonemic contrast, namely the place of articulation. The generation of these stimuli is described in Aerts et al. (2013). The stimuli were presented by E-Prime 3.0 (Psychology Software Tools, Pittsburgh, PA, USA) and delivered at the same comfortable listening level for all participants by ER1 insert earphones (Etymotic Research). In both oddball paradigms, the standard and deviant stimuli appeared random with a probability of respectively 0.80 and 0.20.

#### Inattentive Oddball Paradigm (MMN)

Phoneme discrimination was evaluated by an inattentive auditory oddball paradigm during which the participants were instructed to ignore the stimuli and to focus on a silent movie (Mickey Mouse). The paradigm consisted of 600 standard stimuli and 150 deviant stimuli with a stimulus onset asynchrony of 500 ms. The total duration of the experiment was approximately 7 minutes.

#### Attentive Oddball Paradigm (P300)

Phoneme categorization was evaluated by an attentive auditory oddball paradigm during which the participants were instructed to press the green button on the Chronos response box (Psychology Software Tools, Pittsburgh, PA, USA) when they heard the deviant stimulus. To focus their attention and to reduce vertical and horizontal eye movements, a white fixation cross was presented on a black background. The experimental paradigm consisted of 160 standard stimuli and 40 deviant stimuli with a stimulus onset asynchrony of 2,000 ms. The experimental block was preceded by a training block of 16 standard and four deviant stimuli. The total duration of the experiment was approximately 8 minutes.

### EEG Recording

EasyCap electrode caps (Brain Products, Munich, Germany) were used to record continuous EEG at 32 electrode sites in the HC group and 126 electrode sites in the PPA group. AFz was used as the ground electrode and FCz as the online reference electrode. The electrode impedances were kept below 10 kΩ by using an abrasive electrolyte gel (Abralyt 2000, EasyCap). The preparation of the EEG registration in the patient group was done by two experienced researchers simultaneously since in these participants a cap with 126 electrodes was used. Data were collected with a BrainVision BrainAmp amplifier (Brain Products, Munich, Germany) and were continuously digitized with a sampling frequency of 500 Hz. The data were recorded with the BrainVision Recorder software (Brain Products, Munich, Germany).

### ERP Data Analysis

BrainVision Analyzer 2 (Brain Products, Munich, Germany) was used for the offline EEG analysis. First, the training block of the P300 paradigm was removed and the bad electrode channels were disabled. The online reference electrodes and the electrodes of interest were not disabled in any of the participants. Second, IRR filtering (low cut-off 0.3 Hz, high cut-off 30 Hz, slope 12 dB/oct) and a notch filter at 50 Hz was applied. Based on the independent component analysis (ICA), the eye blinks and horizontal eye movements were removed, and subsequently, the disabled channels were interpolated. A new reference, the average of the mastoid electrodes (TP9 and TP10), was applied to the data. Next, the responses elicited by the standard stimuli and the responses elicited by the deviant stimuli were segmented separately. The epochs of the inattentive oddball paradigm were 600 ms from 100 ms before the onset of the stimulus to 500 ms after the onset of the stimulus. The epochs of the attentive oddball paradigm were 1,500 ms from 300 ms before the onset of the stimulus to 1,200 ms after the onset of the stimulus. Baseline correction was applied using a pre-stimulus window of respectively 100 ms and 300 ms for the inattentive and attentive oddball paradigms. Subsequently, artifact rejection was applied automatically with the following settings: maximum gradient criterion of 75 μV, minimal-maximal amplitude criterion of 100 μV, maximum difference criterion of 150 μV, and low activity criterion of 0.5 μV during 100 ms. Finally, the standard and deviant trials were averaged separately. At least 85% of the trials were included in the averaged ERPs in all participants. The button press accuracies of the P300 paradigm were also collected but both the trials with a correct and incorrect button press response were included in the analysis. The difference waves were computed by subtracting the averaged ERPs elicited by the standard stimuli from the averaged ERPs elicited by the deviant stimuli.

Two main outcome variables, the mean amplitude and onset latency, were extracted from the difference waves from both the inattentive and attentive oddball paradigm. For the MMN and the P300, elicited respectively by the inattentive and attentive oddball paradigm, the average voltage over three specified measurement windows was computed (Luck, 2005). Based on previous research of the MMN and the P300 components (Luck and Kappenman, 2012), visual inspection of the topographic distribution of the components in the HC group, and the hypothesis that the MMN and P300 components might be delayed or prolonged in patients with PPA, the following measurement windows were defined for the MMN: 150–250, 250–350, 350–450 ms, and for the P300: 350–550, 550–750, 750–950 ms. For the onset latency, the negative area over the time window 150–450 ms for the MMN and the positive area over the time window 350–950 ms for the P300 under the ERP difference waveforms were computed. The time point that divided the first 25% of the area from the last 75% of the area was defined as the onset latency, or 25% fractional area latency (Luck, 2005). The mean of the mean amplitudes and onset latencies was calculated for different electrode subsets namely the frontal (F3, F4, Fz), central (C3, C4, Cz), parietal (P3, P4, Pz), left (F3, C3, P3), midline (Fz, Cz, Pz), and right electrode sites (F4, C4, P4).

### Interpretation ERPs

Since PPA consists of a heterogeneous group of clinical syndromes and the included patients were also heterogeneous concerning the clinical variant and disease stage, the ERPs were interpreted at the single-subject level. The analysis on the single-subject level is particularly interesting to investigate the applicability of ERPs in the diagnostic process of PPA and its variants in clinical practice. In clinical practice, the presence of ERP components has been traditionally assessed by visual inspection. Statistical methods for single-subject analyses of ERPs have also been proposed but contradictory results were reported for the different approaches (Kallionpää et al., 2019). Kallionpää et al. (2019) compared five methods to interpret ERPs at the single-subject level and they concluded that one method alone may not be sufficient to decide on the presence or absence of an ERP component. They propose a combination of two or more different methods to interpret the ERPs. In this study, we combined two methods that are easy to use in clinical practice to possibly improve the diagnostic process.

The first method we applied was a visual inspection of the single-subject averaged ERPs for both paradigms in the patients as well as in the HCs. This method is part of the clinical routine used in many institutions and is sensitive to detect differences in topography, latency, and morphology of the components. For the pre-attentive oddball paradigm, the difference waveforms at the different electrode subsets and the topographic distribution map were presented in figures. For the attentive oddball paradigm, the average waveforms elicited by the standard and deviant stimuli were presented separately on the same figure at the different electrode subsets. The difference waveforms and the topographic distribution map were also shown in figures. The figures of the patients and the HCs were presented in a randomized order to two raters, who independently evaluated whether the MMN and P300 were present. To compare the figures of each patient with the HC group, the averaged ERPs of the HC group were also plotted in the same manner as the ERPs of the individual patients. For the patients, each rater also evaluated if the component was delayed, prolonged, or showed an accelerated decay in comparison to the components of the HC group. The component was defined to be present if both raters agreed on it (Kallionpää et al., 2019).

After visual inspection, the individual amplitudes and latencies of the components that were concluded to be present in the patients with PPA were compared to the HC group by converting their raw values into standardized *Z*-scores (Crawford et al., 2006). These *Z*-scores were calculated based on the means and standard deviations of the mean amplitudes and onset latencies at the various electrode subsets of both paradigms in the HC group. Since this study is the first to evaluate the MMN and P300 components elicited by linguistic stimuli in PPA, our hypotheses were based on studies of nondegenerative aphasic patients (Csépe et al., 2001; Ilvonen et al., 2003, 2004; Becker and Reinvang, 2007; Robson et al., 2014; Aerts et al., 2015; Dejanovic et al., 2015). Our hypotheses were directional for the onset latency and non-directional for the mean amplitude of both the MMN and P300. Concerning the MMN, the mean amplitudes might be reduced (positive *Z*-scores) or increased (negative *Z*-scores) and the onset latencies might be delayed (positive *Z*-scores) in patients with PPA in comparison to the HC group. Regarding the P300, the mean amplitudes might be reduced (negative *Z*-scores) or increased (positive *Z*-scores) and the onset latencies might be delayed (positive *Z*-scores) in patients with PPA in comparison to the HC group. Since we do not know which *Z*-scores are clinically significant, we consider *Z*-scores ≥ 1.28 (α ≤ 0.10) as impaired in comparison to the HC group.

## Results

**Figure 1:**
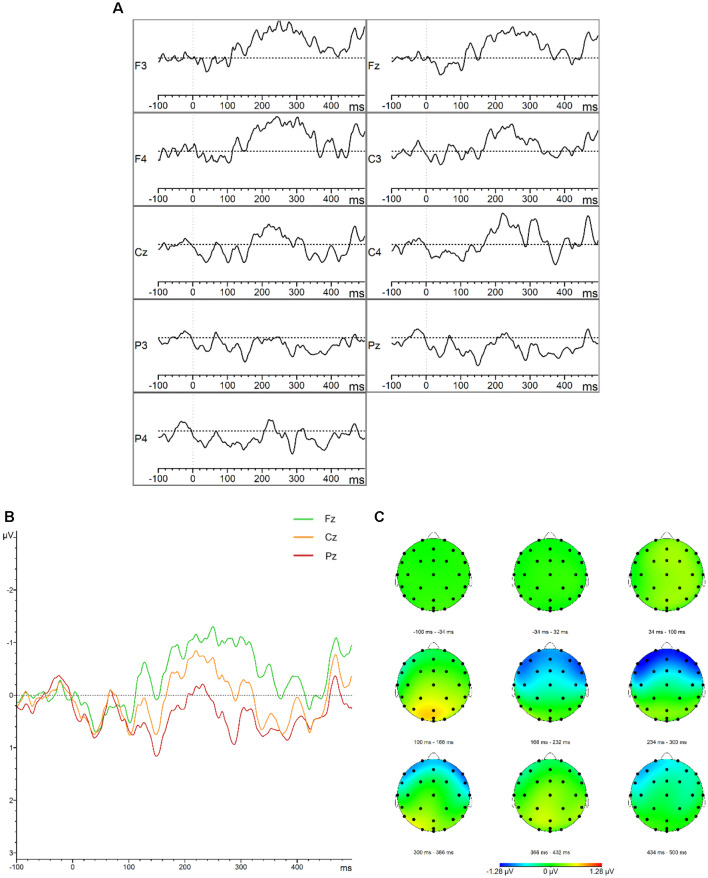
Mismatch negativity (MMN). **(A)** Grand average difference waveforms (deviant–standard condition) for the control group at the frontal, central, parietal, left, midline, and right electrode sites. **(B)** Grand average difference waveforms (deviant–standard condition) for the control group at the midline electrode sites. **(C)** Topographic distribution of the grand average difference waveforms for the control group.

**Figure 2:**
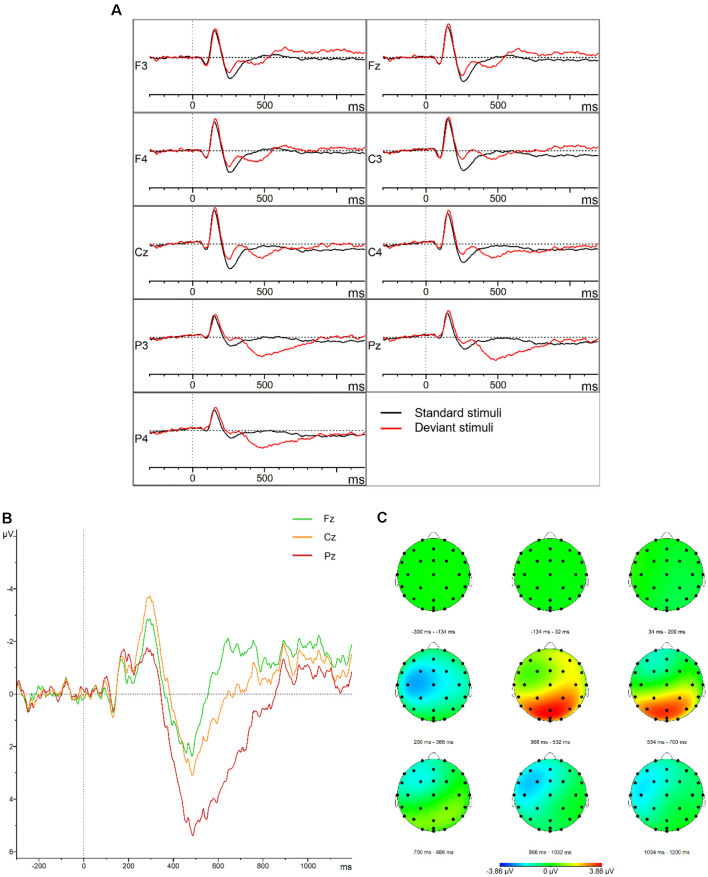
P300. **(A)** Grand average waveforms for the control group elicited by the standard and deviant stimuli at the frontal, central, parietal, left, midline, and right electrode sites. **(B)** Grand average difference waveforms (deviant–standard condition) for the control group at the midline electrode sites. **(C)** Topographic distribution of the grand average difference waveforms for the control group.

The descriptive statistics of the mean amplitudes and onset latencies of the MMN and P300 difference waves for the HC group are presented in Tables s 1 and 2, respectively. The grand average waveforms and topographic distributions of the MMN and P300 components of the HC group are shown in Figures 1 and 2, respectively. The mean accuracy of the button press responses of the HC group during the P300 experiment was 96% (*M* = 192, SD = 18.19). The button press response was correct in 97.42% of the trials with the standard stimuli (*M* = 155.87, SD = 12.54) and in 90.33% of the trials with the deviant stimuli (*M* = 36.13, SD = 7.02). Visual inspection of each healthy participant showed that the MMN was present in 29 of the 30 HCs and the P300 was present in 28 of the 30 HCs.

**Table 1:** The descriptive statistics of the mean amplitudes (in μV) and onset latencies (in ms) of the mismatch negativity (MMN) difference waves for the control group at the frontal, central, parietal, left, midline, and right electrode sites.

	Electrode	Mean	SD	95% CI	Min	Max	IQR
**Mean amplitude 150–250 ms**	F	−1.60	0.76	−1.31 to −1.88	−1.88	−3.23	0.12
	C	−1.34	0.74	−1.07 to −1.62	−1.62	−3.35	0.17
	P	−0.78	0.60	−0.55 to −1.00	−1.00	−1.82	0.34
	L	−1.19	0.57	−0.97 to −1.40	−1.40	−2.40	−0.09
	M	−1.33	0.72	−1.07 to −1.60	−1.60	−2.82	0.25
	R	−1.20	0.69	−0.94 to −1.46	−1.46	−2.69	0.19
**Mean amplitude 250–350 ms**	F	−1.03	0.81	−0.73 to −1.34	−1.34	−3.07	0.97
	C	−0.85	0.80	−0.55 to −1.15	−1.15	−2.83	0.93
	P	−0.49	0.66	−0.24 to −0.74	−0.74	−1.61	1.11
	L	−0.77	0.63	−0.54 to −1.01	−1.01	−2.16	0.94
	M	−0.82	0.79	−0.52 to −1.11	−1.11	−2.37	0.83
	R	−0.79	0.73	−0.51 to −1.06	−1.06	−2.50	0.76
**Mean amplitude 350–450 ms**	F	−0.32	0.82	−0.01 to −0.62	−0.62	−2.56	1.84
	C	−0.30	0.81	0.00 to −0.60	−0.60	−2.65	1.66
	P	−0.37	0.70	−0.11 to −0.63	−0.63	−1.75	1.12
	L	−0.28	0.67	−0.03 to −0.53	−0.53	−1.80	1.70
	M	−0.35	0.80	−0.05 to −0.65	−0.65	−2.05	1.56
	R	−0.36	0.75	−0.08 to −0.64	−0.64	−2.49	1.37
**Onset latency**	F	208.69	16.46	202.54–214.84	202.54	187.33	241.33
	C	211.40	16.04	205.41–217.39	205.41	187.33	247.33
	P	216.44	20.99	208.61–224.28	208.61	180.00	267.33
	L	212.82	16.81	206.54–219.10	206.54	182.67	244.67
	M	211.71	17.81	205.06–218.36	205.06	178.67	250.67
	R	212.00	15.94	206.05–217.95	206.05	180.67	252.67

*Abbreviations: F, frontal; C, central; P, parietal; L, left; M, midline; R, right; SD, standard deviation; CI, confidence interval; Min, minimum; Max, maximum; IQR, interquartile range*.

**Table 2:** The descriptive statistics of the mean amplitudes (in μV) and onset latencies (in ms) of the P300 difference waves for the control group at the frontal, central, parietal, left, midline, and right electrode sites.

	Electrode	Mean	SD	95% CI	Min	Max	IQR
**Mean amplitude 350–550 ms**	F	1.27	2.98	0.16–2.38	−4.93	7.06	4.90
	C	1.32	3.47	0.02–2.61	−6.55	8.76	3.71
	P	3.24	3.84	1.81–4.67	−4.74	11.52	4.59
	L	1.55	2.93	0.46–2.65	−4.78	8.02	2.49
	M	2.10	3.44	0.81–3.38	−5.54	9.50	3.81
	R	2.17	3.05	1.04–3.31	−5.90	8.83	3.35
**Mean amplitude 550–750 ms**	F	−1.11	2.36	−1.99 to −0.23	−6.43	2.62	3.21
	C	0.37	2.73	−0.65–1.39	−6.76	4.76	3.34
	P	2.72	2.91	1.63–3.80	−3.49	9.16	3.85
	L	0.40	2.16	−0.40–1.21	−5.38	4.04	2.67
	M	0.65	2.49	−0.28–1.58	−5.08	4.42	3.27
	R	0.92	2.12	0.13–1.71	−3.69	4.16	1.77
**Mean amplitude 750–950 ms**	F	−1.29	2.21	−2.11 to −0.46	−7.21	3.33	2.95
	C	−0.82	2.05	−1.58 to −0.05	−5.32	3.81	2.43
	P	0.07	2.23	−0.76–0.90	−3.88	5.10	2.86
	L	−1.12	1.74	−1.77 to −0.47	−4.87	2.42	1.69
	M	−0.79	2.18	−1.60–0.03	−4.83	4.68	2.68
	R	−0.13	1.62	−0.74–0.48	−4.02	3.86	1.81
**Onset latency**	F	491.51	121.23	446.24–536.78	365.33	834.67	98.00
	C	472.27	69.75	446.22–498.31	352.00	674.67	100.17
	P	494.42	80.78	464.26–524.58	367.33	818.00	54.33
	L	487.58	85.53	455.64–519.51	363.33	818.00	72.67
	M	489.53	88.84	456.36–522.71	365.33	833.33	84.67
	R	481.09	63.26	457.47–504.71	366.00	676.00	50.50

*Abbreviations: F, frontal; C, central; P, parietal; L, left; M, midline; R, right; SD, standard deviation; CI, confidence interval; Min, minimum; Max, maximum; IQR, interquartile range*.

The electrophysiological results of each included patient with PPA were compared to the results of the HC group. A summary of the neurological, language, speech, and electrophysiological examinations of each included patient with PPA is presented in the Results section. The demographic characteristics, the scores on the MoCA, and the PTA of each patient can be found in Table 3. For each included patient, the results of the CAT-NL are presented in Table 4 and the results of the DIAS in Table 5. The figures of the average waveforms and the topographic distribution maps of both the MMN and the P300 can be found in the Results section for each patient. In the Supplementary Material, the raw values and the corresponding *Z*-scores of the mean amplitudes and onset latencies of the MMN and the P300 are presented.

**Table 3:** Demographic characteristics of the included patients with primary progressive aphasia (PPA).

Case	1	2	3	4	5	6	7	8
Variant	NFV	NFV	NFV	NOS	LV	LV	SV	SV
Stage of PPA	early	early	late	early	early	late	early	late
Sex	M	M	F	F	M	F	M	M
Handedness	R	R	R	R	L	L	R	R
Education (years)	15	12	12	12	14	10	12	11
Age at onset (years; months)	63	64;4	75	68	64	52	70	67
Age first consultation at UZ Ghent (years; months)	64	65;6	77;7	69;9	67;3	53;4	72;7	68;1
Age at testing (years; months)	66	65;9	79;7	70	68;9	54;5	74;3	70;6
MoCA total score (30)	28	25	16	23	27	12	27	17
PTA best ear (dB HL)	18.8	15	32.5	16.3	25	15	15	30

*Abbreviations: NFV, nonfluent variant; LV, logopenic variant; SV, semantic variant; NOS, not otherwise specified; M, male; F, female; R, right; L, left; UZ Ghent, Ghent University Hospital; MoCA, Montreal Cognitive Assessment; PTA, pure tone average (average air conduction hearing thresholds at 500 Hz, 1,000 Hz, 2,000 Hz, and 4,000 Hz)*.

**Table 4:** Raw scores and corresponding C-scores of all subtests of the CAT-NL for each included patient with PPA.

	Case	1	2	3	4	5	6	7	8
**Subtest**	**Total**	**Raw scores (C-scores)**							
**Cognitive screening**		30 (5)	37 (9)	**24 (4)**	33 (7)	39 (10)	**22 (3)**	38 (10)	**28 (5)**
Line bisection		1 (4)	0 (6)	−0.5 (5)	0 (6)	0 (6)	**3 (2)**	0 (6)	0 (6)
Semantic memory	10	10 (6)	10 (6)	**7 (3)**	10 (6)	10 (6)	**7 (3)**	10 (6)	**3 (1)**
Fluency		16 (6)	22 (8)	**5 (4)**	**11 (5)**	46 (10)	**10 (5)**	32 (9)	**13 (6)**
Recognition memory	10	10 (5)	10 (5)	10 (5)	9 (4)	10 (5)	**6 (3)**	9 (4)	**7 (3)**
Ideational praxis	12	**8 (3)**	11 (6)	**6 (3)**	11 (6)	11 (6)	11 (6)	12 (7)	11 (6)
Arithmetic	6	6 (6)	6 (6)	4 (4)	6 (6)	6 (6)	**2 (3)**	6 (6)	6 (6)
**Comprehension**		18 (10)	18 (10)	**10 (5)**	14 (7)	17 (9)	**8 (4)**	17 (9)	**10 (5)**
**Spoken total**	66	65 (9)	66 (9)	**54 (5)**	64 (8)	62 (7)	**46 (4)**	65 (9)	**55 (5)**
Spoken words	30	30 (7)	30 (7)	30 (7)	30 (7)	29 (7)	**24 (4)**	29 (7)	**24 (4)**
Spoken sentences	32	31 (8)	32 (8)	**22 (4)**	30 (7)	30 (7)	**20 (4)**	32 (8)	28 (6)
Spoken paragraphs	4	4 (6)	4 (6)	2 (4)	4 (6)	3 (5)	2 (4)	4 (6)	3 (5)
**Written total**	62	61 (9)	61 (9)	**49 (5)**	55 (6)	62 (10)	**42 (4)**	60 (8)	**49 (5)**
Written words	30	30 (6)	30 (6)	30 (6)	30 (6)	30 (6)	28 (5)	30 (6)	**20 (3)**
Written sentences	32	31 (9)	31 (9)	**19 (4)**	25 (6)	32 (9)	**14 (3)**	30 (8)	29 (7)
**Production**		32 (9)	30 (8)	27 (7)	29 (8)	31 (9)	**22 (5)**	31 (9)	**23 (6)**
**Repetition total**	61	59 (8)	57 (7)	**48 (5)**	56 (7)	**46 (5)**	54 (6)	58 (7)	57 (7)
Words	32	32 (6)	32 (6)	28 (5)	32 (6)	28 (5)	30 (5)	32 (6)	32 (6)
Complex words	6	6 (6)	6 (6)	6 (6)	6 (6)	4 (5)	6 (6)	6 (6)	6 (6)
Nonwords	10	10 (7)	9 (6)	6 (5)	10 (7)	4 (4)	10 (7)	10 (7)	10 (7)
Numbers	7	5 (6)	4 (5)	4 (5)	4 (5)	5 (6)	4 (5)	5 (6)	4 (5)
Sentences	6	6 (7)	6 (7)	**4 (5)**	**4 (5)**	5 (6)	**4 (5)**	5 (6)	5 (6)
**Naming total**		71 (8)	77 (8)	**55 (6)**	62 (7)	104 (10)	**52 (6)**	83 (9)	**34 (4)**
Objects	48	45 (9)	45 (9)	41 (8)	42 (8)	48 (10)	36 (6)	41 (8)	**11 (4)**
Actions	10	10 (7)	10 (7)	9 (6)	10 (7)	10 (7)	**6 (5)**	10 (7)	10 (7)
**Reading aloud total**	70	70 (8)	68 (7)	70 (8)	68 (7)	70 (8)	64 (6)	70 (8)	63 (6)
Words	48	48 (7)	46 (6)	48 (7)	48 (7)	48 (7)	48 (7)	48 (7)	45 (6)
Complex words	6	6 (6)	6 (6)	6 (6)	6 (6)	6 (6)	**4 (5)**	6 (6)	**4 (5)**
Function words	6	6 (5)	6 (5)	6 (5)	6 (5)	6 (5)	6 (5)	6 (5)	6 (5)
Nonwords	10	10 (7)	10 (7)	10 (7)	8 (6)	10 (7)	**6 (5)**	10 (7)	8 (6)
**Writing total**	83	82 (8)	81 (8)	82 (8)	81 (8)	82 (8)	**49 (4)**	77 (7)	76 (6)
Copying	32	31 (6)	31 (6)	31 (6)	31 (6)	31 (6)	**17 (4)**	31 (6)	31 (6)
Naming objects	23	23 (7)	22 (7)	23 (7)	22 (7)	23 (7)	18 (5)	22 (7)	21 (6)
To dictation	28	28 (7)	28 (7)	28 (7)	28 (7)	28 (7)	**14 (4)**	**24 (5)**	**24 (5)**

*Scores in bold, impaired. CAT-NL, Dutch version of the Comprehensive Aphasia Test*.

**Table 5:** Raw scores of all subtests of the DIAS for each included patient with PPA.

			1	2	3	4	5	6	7	8
**Subtest**	**Total**	**Raw scores**							
**Buccofacial movements**		30	**14**	30	**23**	**29**	30	30	30	30
Characteristic A: improved performance when imitating			0	0	**3**	0	0	**2**	0	0
Characteristic B: articulatory groping			**8**	1	0	1	0	**1**	0	0
**Articulation consonants and vocals**		30	**27**	**28**	30	30	30	**29**	30	30
C1: inconsistent production			**3**	1	0	0	0	1	0	0
C2: worse performance consonants than vocals			1	2	0	0	0	1	0	0
**Diadochokinesis**			**57**	94	−	**75**	164	103	178	122
C3: worse performance alternating sequences than sequential sequences			1.06	0.99	0.79	**0.55**	0.91	**0.65**	0.92	0.83
C4: articulatory groping			**Yes**	**Yes**	No	**Yes**	No	No	No	No
**Articulation words**		264	239	247	261	264	263	260	264	264
C5: initiation problems			0.09	**0.27**	0	0	0	0	0	0
C6: syllable segmentations			**0.5**	**0.83**	**0.17**	0	0	0	0	0
C7: cluster segmentations			**0.2**	**0.2**	0	0	0	0	0	0
C8: articulation complexity effect			**2**	0.30	0	0	0	**1.33**	0	0
**Buccofacial apraxia**			**Yes**	No	Yes*	No	No	Yes*	No	No
**Apraxia of speech**			**Yes**	**Yes**	No	No	No	No	No	No

Abbreviations: C, characteristic; scores in bold, impaired; DIAS, Diagnostic Instrument for Apraxia of Speech; *impaired due to comprehension difficulties.

### Case 1—Early—Stage NFV

*Diagnosis—*Case 1 met the diagnostic criteria for the clinical diagnosis of the NFV (Gorno-Tempini et al., 2011). Effortful speech, agrammatism, AOS, and word-finding difficulties were the key characteristics of his spontaneous speech. His AOS was confirmed by the DIAS, but no scores outside the normative range were found in the results of the CAT-NL. The clinical neurological examination showed a mild extrapyramidal syndrome and apraxia (left more than right). Neuroimaging results revealed frontal and temporal corticosubcortical atrophy (predominant left frontal) on MRI and mild bilateral frontal hypometabolism on FDG-PET. No arguments for AD were found by CSF biomarker examination.

**Figure 3:**
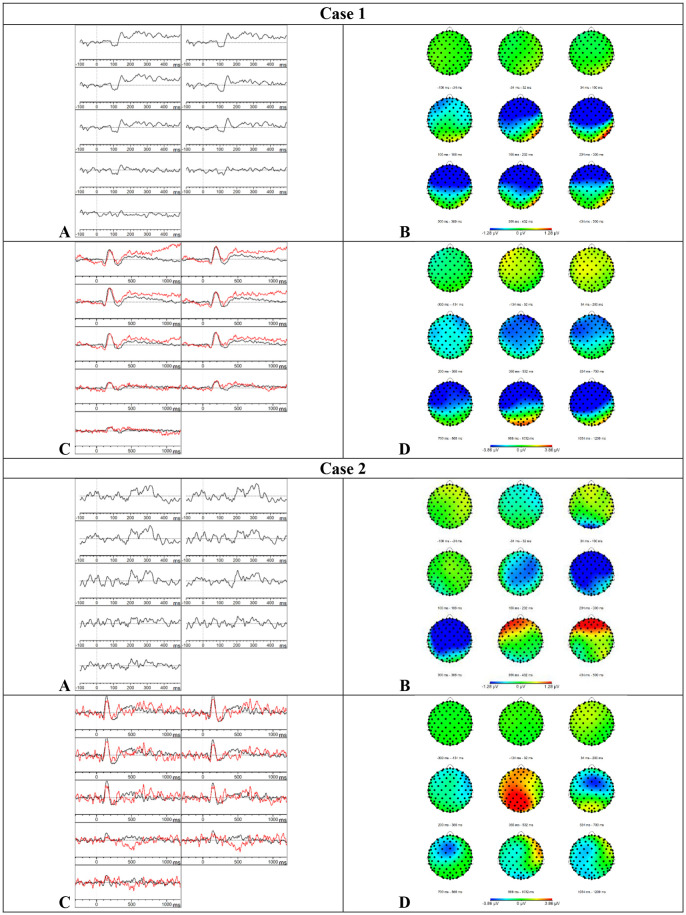
Results case 1 and 2. **(A)** Difference waveforms elicited by the MMN paradigm at F3, Fz, F4, C3, Cz, C4, P3, Pz, and P4 (left to right). **(B)** Topographic distribution of the difference waveforms elicited by the MMN paradigm. **(C)** Average waveforms elicited by the standard (black) and deviant (red) stimuli of the P300 paradigm at F3, Fz, F4, C3, Cz, C4, P3, Pz, and P4 (left to right). **(D)** Topographic distribution of the difference waveforms elicited by the P300 paradigm.

*Results* *MMN—*Visual inspection of Figure 3 showed that the MMN was present but that the component was prolonged in comparison to the MMN of the HC group. Based on the results of the *Z*-scores, the mean amplitude was increased at the frontal electrode sites in time windows 250–350 ms (*Z* = −2.37) and 350–450 ms (*Z* = −2.41) which confirms the prolongation found by visual inspection. The onset latency was also delayed at the frontal electrode sites (*Z* = 1.66). Furthermore, the mean amplitude was reduced at the parietal electrode sites in the time window 150–250 ms (*Z* = 1.33).

*Results P300*—Based on the visual inspection of Figure 3, we concluded that the P300 was absent. The button press accuracy of case 1 was 85.5%.

### Case 2—Early-Stage NFV

*Diagnosis—*Case 2 met the diagnostic criteria for the clinical diagnosis of the NFV (Gorno-Tempini et al., 2011). A slow speech rate, a reduction of spontaneous speech, short sentences, sporadically telegraphic speech, AOS, and word-finding difficulties were the key characteristics of his spontaneous speech. His AOS was confirmed by the DIAS, but no scores outside the normative range were found in the results of the CAT-NL. The clinical neurological examination showed a right-lateralized, mild extrapyramidal syndrome. The neuroimaging results revealed a normal MRI scan and a mild hypometabolism in the right precuneus on FDG-PET, of unknown significance.

*Results* *MMN—*Visual inspection of Figure 3 showed that the MMN was present. The *Z*-scores showed a delay of the component at the frontal (*Z* = 1.86) and left (*Z* = 1.46) electrode sites and the mean amplitude was reduced at the frontal electrode sites (*Z* = 1.29) in the time window 150–250 ms. In the subsequent time window 250–350 ms, the mean amplitude was increased at the frontal (*Z* = −1.63) and left (*Z* = −1.42) electrode sites. In the time window 350–450 ms, the mean amplitude was reduced at the frontal electrode sites (*Z* = 1.5) which suggests an accelerated decay.

*Results P300*—Visual inspection of Figure 3 showed that the P300 was present. Furthermore, the P300 showed an accelerated decay in comparison to the P300 of the HC group. The mean amplitudes and onset latencies did not show *Z*-scores ≥ 1.28. The button press accuracy of case 2 was 100%.

### Case 3—Late-Stage NFV

*Diagnosis—*Case 3 was initially diagnosed with the NFV (Gorno-Tempini et al., 2011). At the time of testing, her spontaneous speech was characterized by empty speech, perseverations, echolalia, confabulations, word-finding difficulties, semantic neologisms, semantic paraphasias, and stereotypical sentences. Based on the results of the MoCA, she also presented with executive dysfunctions, attention deficits, and short–term memory deficits. Scores below the normative range were found in the cognitive screening, sentence comprehension subtest, repetition of sentences subtest, and naming subtests of the CAT-NL. A left fronto-insular hypometabolism was found on FDG-PET and a low beta-amyloid concentration was found by CSF biomarker examination.

**Figure 4:**
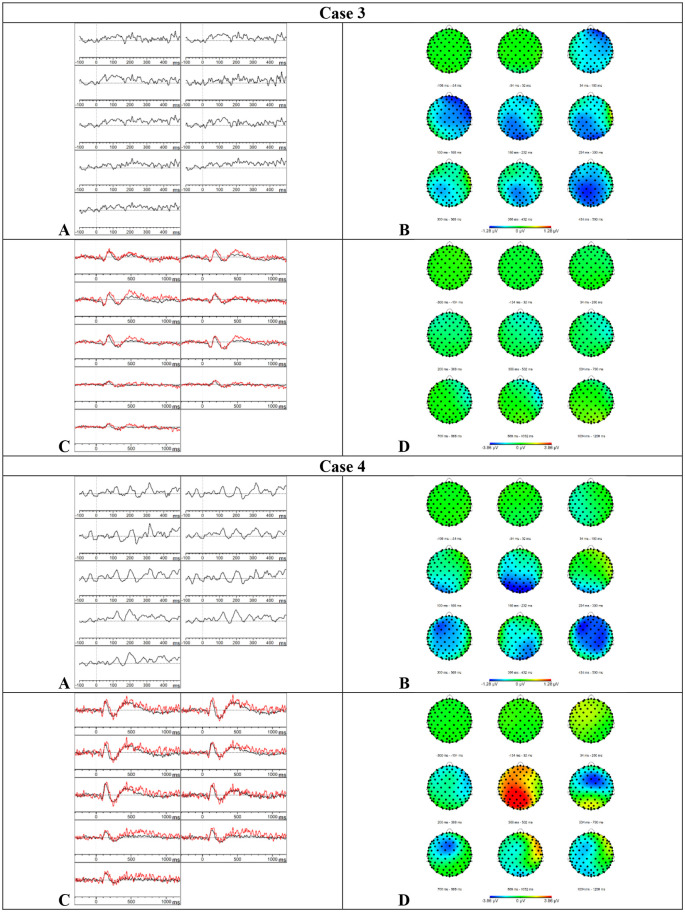
Results case 3 and 4. **(A)** Difference waveforms elicited by the MMN paradigm at F3, Fz, F4, C3, Cz, C4, P3, Pz, and P4 (left to right). **(B)** Topographic distribution of the difference waveforms elicited by the MMN paradigm. **(C)** Average waveforms elicited by the standard (black) and deviant (red) stimuli of the P300 paradigm at F3, Fz, F4, C3, Cz, C4, P3, Pz, and P4 (left to right). **(D)** Topographic distribution of the difference waveforms elicited by the P300 paradigm.

*Results* *MMN—*Based on the visual inspection of Figure 4, we concluded that the MMN was absent.

*Results P300*–Based on the visual inspection of Figure 4, we concluded that the P300 was absent. It is possible that case 3 did not understand the task because she had language comprehension deficits and she did not press the button during the whole paradigm.

### Case 4—Early-Stage PPA-NOS

*Diagnosis—*The spontaneous speech of case 4, at the time of testing, was characterized by word-finding difficulties, phonological and semantic paraphasias, a reduction of spontaneous speech, paragrammatic errors, interrupted sentences, sporadic telegraphic speech, and mild AOS. Scores outside the normative range were found in the fluency and sentence repetition subtests of the CAT-NL. Based on these results, case 4 met one core feature and two supportive features of the NFV and two core features and three supportive features of the LV (Gorno-Tempini et al., 2011). The neuroimaging results revealed mild atrophy at the left frontal and temporal operculum on MRI and a left fronto-insular hypometabolism on FDG-PET which is consistent with the NFV. The clinical phenotype remained unclear 6 months later. At that time, case 4 also showed sentence comprehension deficits and more paragrammatic and agrammatic errors in her spontaneous speech. She met the two core features of both the NFV and the LV, three supportive features of the NFV, and two supportive features of the LV. Consequently, we describe case 4 as PPA-NOS (not otherwise specified).

*Results MMN—*Based on the visual inspection of Figure 4, we concluded that the MMN was absent.

*Results P300*–Based on the visual inspection of Figure 4, we concluded that the P300 was absent. Case 4 showed a button press accuracy of 0% for the deviant stimuli. During the registration of the attentive oddball paradigm, she communicated that she did not hear differences between the stimuli but that she did understand the task.

### Case 5—Early-Stage LV

*Diagnosis—*Case 5 met the diagnostic criteria for the clinical diagnosis of the LV (Gorno-Tempini et al., 2011). Word-finding difficulties, circumlocutions, phonological paraphasias, semantic paraphasias, and unfinished sentences were the key characteristics of his spontaneous speech. Scores below the normative range were only found in the repetition subcategory of the CAT-NL (mainly in the subtests of complex words and sentences). Neuroimaging results revealed a normal MRI scan and a hypometabolism in the right precuneus on FDG-PET. CSF biomarker profile was not compatible with Alzheimer’s disease.

**Figure 5:**
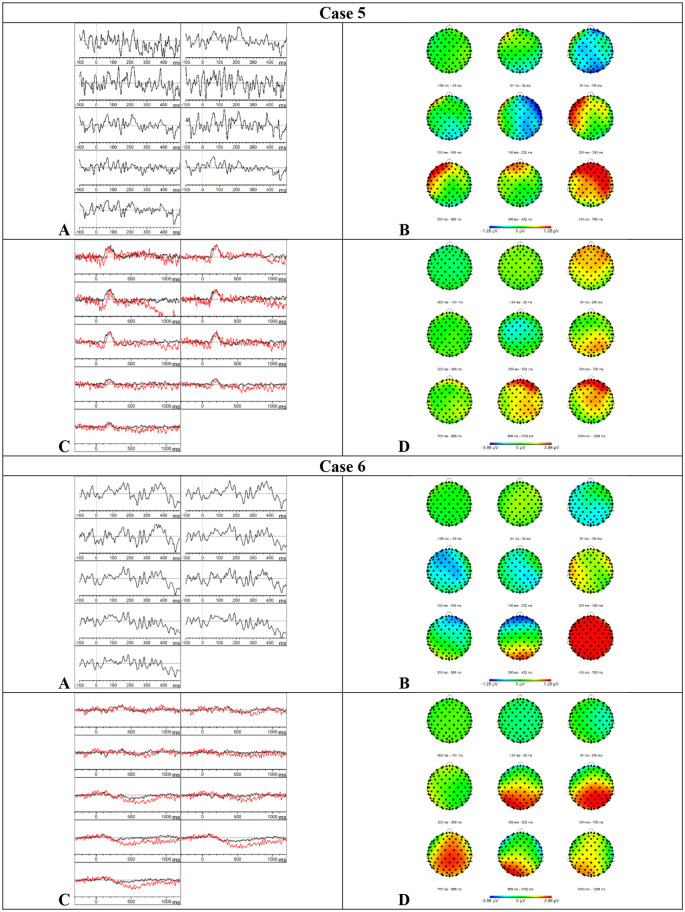
Results case 5 and 6. **(A)** Difference waveforms elicited by the MMN paradigm at F3, Fz, F4, C3, Cz, C4, P3, Pz, and P4 (left to right). **(B)** Topographic distribution of the difference waveforms elicited by the MMN paradigm. **(C)** Average waveforms elicited by the standard (black) and deviant (red) stimuli of the P300 paradigm at F3, Fz, F4, C3, Cz, C4, P3, Pz, and P4 (left to right). **(D)** Topographic distribution of the difference waveforms elicited by the P300 paradigm.

*Results MMN—*Visual inspection of Figure 5 showed that the MMN component was present. Furthermore, an accelerated decay in comparison to the MMN of the HC group was found. The mean amplitudes and onset latencies did not show *Z*-scores ≥ 1.28.

*Results P300*—Based on the visual inspection of Figure 5, we concluded that the P300 component was present with a delayed latency. Furthermore, the component was prolonged in comparison to the results of the HC group. This delay and prolongation were confirmed by the *Z*-scores. The onset latency was delayed at central (*Z* = 2.73), parietal (*Z* = 2.15), left (*Z* = 1.77), midline (*Z* = 1.91), and right (*Z* = 2.99) electrode sites. The mean amplitude was increased in the time window 750–950 ms at the central (*Z* = 1.36), left (*Z* = 1.31), and right (*Z* = 1.32) electrode sites. Surprisingly, the button press accuracy was only 24%.

### Case 6—Late-Stage LV

*Diagnosis—*Case 6 was initially diagnosed with the LV (Gorno-Tempini et al., 2011). At the time of testing, empty speech, word-finding difficulties, neologisms, circumlocutions, phonological and semantic paraphasias, confabulations, stereotypical sentences, short sentences, and unfinished, interrupted sentences were the key characteristics of her spontaneous speech. The presence of visuospatial deficits, executive dysfunctions, attention deficits, agnosia, apraxia, and short-term memory deficits was shown by the neurological examination and the MoCA. Scores outside the normative range were found in the cognitive screening, language comprehension, and language production subtests of the CAT-NL. MRI showed corticosubcortical atrophy (right more than left) with dilation of the occipital and temporal horn of the lateral ventricle. A widespread bilateral (right more than left) temporal, parietal, and occipital hypometabolism was found on FDG-PET. The left-handedness of case 6 might explain the right-lateralized atrophy. CSF biomarker profile showed a normal tau and a low beta-amyloid concentration. No genetic mutations were found in the genes C9orf72, MAPT, VCP, and GRN.

*Results MMN—*Based on the visual inspection of Figure 5, we concluded that the MMN was minimally present with a severe delay. Consistently, the mean amplitudes in the initial time window (150–250 ms) were reduced at the frontal (*Z* = 1.78), left (*Z* = 1.56), midline (*Z* = 1.36), and right (*Z* = 1.36) electrode sites. The mean amplitude was also reduced in the time window 250–350 ms at the left electrode sites (*Z* = 1.61) and in the time window 350–450 ms at the parietal electrode sites (*Z* = 1.68).

*Results P300*—Visual inspection of Figure 5 showed that the P300 component was present but prolonged in comparison to the P300 component in the HC group. Accordingly, the mean amplitude was increased in the time window 750–950 ms at the parietal (*Z* = 1.3), left (*Z* = 1.58), and midline (*Z* = 1.49) electrode sites. Furthermore, the onset latency was delayed at the frontal electrode sites (*Z* = 1.3). The button press accuracy of case 6 was 66%.

### Case 7—Early-Stage SV

*Diagnosis—*Case 7 met the two core diagnostic criteria and two of the four supportive criteria of the SV (Gorno-Tempini et al., 2011). He complained about word comprehension deficits and word-finding difficulties but the CAT-NL did not show any scores outside the normative range. Word-finding difficulties, circumlocutions, semantic paraphasias, neologisms, and logorrhea were the key characteristics of his spontaneous speech. The neuroimaging results revealed a predominant bilateral anterior temporal lobe atrophy on MRI and hypometabolism on FDG-PET.

**Figure 6:**
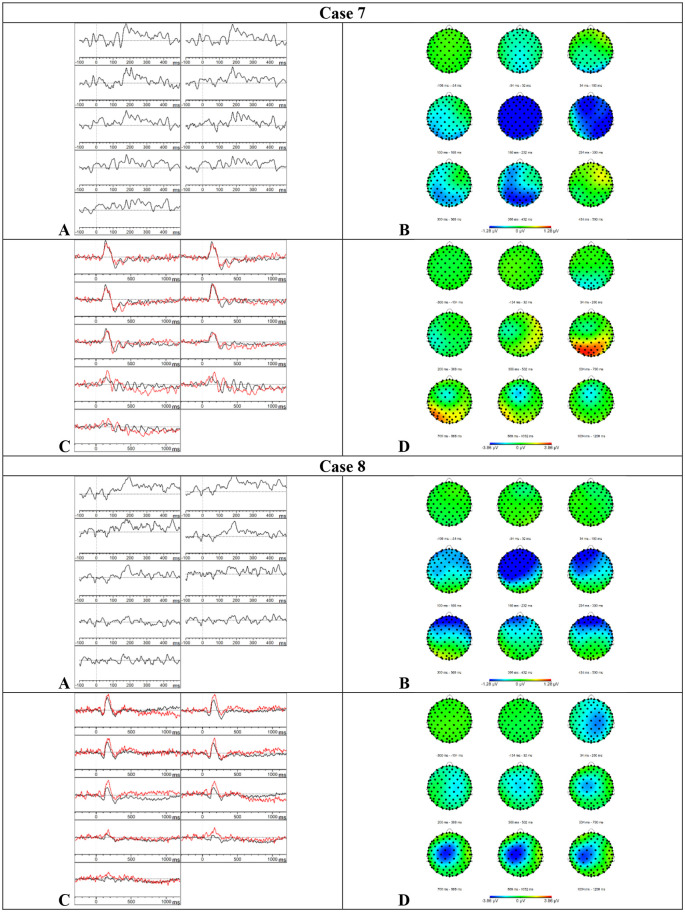
Results case 7 and 8. **(A)** Difference waveforms elicited by the MMN paradigm at F3, Fz, F4, C3, Cz, C4, P3, Pz, and P4 (left to right). **(B)** Topographic distribution of the difference waveforms elicited by the MMN paradigm. **(C)** Average waveforms elicited by the standard (black) and deviant (red) stimuli of the P300 paradigm at F3, Fz, F4, C3, Cz, C4, P3, Pz, and P4 (left to right). **(D)** Topographic distribution of the difference waveforms elicited by the P300 paradigm.

*Results MMN—*Visual inspection of Figure 6 showed that the MMN was present. The mean amplitudes and onset latencies did not show *Z*-scores ≥ 1.28.

*Results P300*—Visual inspection of Figure 6 showed that the P300 might be present at various central and parietal electrode sites but these results should be interpreted with caution because the data showed a lot of alpha activity. The mean amplitudes and onset latencies did not show *Z*-scores ≥ 1.28. Accordingly, the button press accuracy was 96.5%.

### Case 8—Late-Stage SV

*Diagnosis—*Case 8 was initially diagnosed with the SV (Gorno-Tempini et al., 2011). At the time of testing, word-finding difficulties, empty speech, circumlocutions, and semantic paraphasias were the key characteristics of his spontaneous speech. Scores below the normative range were found in the subtests semantic memory, fluency, recognition memory, spoken and written word comprehension, naming, reading aloud complex words, and writing to the dictation of the CAT-NL. The neurological and neuropsychological examinations also showed the presence of prosopagnosia, deficits in short-term memory, attention deficits, executive dysfunctions, and a loss of interest and initiative. Neuroimaging results revealed a left temporal and hippocampal atrophy on MRI and a hypometabolism on the left anterior temporal lobe on SPECT. CSF biomarker profile was not compatible with Alzheimer’s disease.

*Results MMN—*Visual inspection of Figure 6 showed that the MMN was present. The mean amplitudes and onset latencies did not show *Z*-scores ≥ 1.28.

*Results P300*—Based on the visual inspection of Figure 6, we concluded that the P300 component was absent. Surprisingly, the button press accuracy was 97%.

### Summary Patients with PPA

In Table 6, the electrophysiological results of the MMN and P300 paradigms were summarized for each included patient with PPA.

**Table 6:** Summary of the electrophysiological results of the MMN and P300 experiments for each included patient with PPA.

Case	Variant	MMN	P300	Button press accuracy P300
1	Early NFV	Present F: delayed, ↑ MA, and prolonged P: MA ↓	Absent	Standard: 144/160 Deviant: 27/40
2	Early NFV	Present F, L: delayed, MA ↑ F: accelerated decay	Present Accelerated decay	Standard: 160/160 Deviant: 40/40
3	Late NFV	Absent	Absent (! Comprehension task)	Standard: 160/160 Deviant: 0/40
4	Early PPA-NOS	Absent		Standard: 160/160 Deviant: 0/40
5	Early LV	Present
		Accelerated decay	Present C, P, L, M, R: delayed C, L, R: prolonged	Standard: 39/160 Deviant: 9/40
6	Late LV	Present F, L, M, R: MA ↓ Delayed	Present F: delayed P, L, M: prolonged	Standard: 114/160 Deviant: 18/40
7	Early SV	Present	Present but data with much alpha activity	Standard: 159/160 Deviant: 36/40
8	Late SV	Present	Absent	Standard: 160/160 Deviant: 34/40

*Abbreviations: NFV, nonfluent variant; LV, logopenic variant; SV, semantic variant; NOS, not otherwise specified; F, frontal electrode sites; C, central electrode sites; P, parietal electrode sites; L, left electrode sites; M, midline electrode sites; R, right electrode sites; MA ↑, increased mean amplitude; MA ↓, decreased mean amplitude*.

## Discussion

This was the first study to investigate the MMN and P300 components in PPA by a preliminary case series. Our results show differences in mean amplitude, onset latency, and/or topographic distribution of the MMN as well as the P300 in the patients with PPA in comparison to the HC group.

The **MMN** component was present in six of the eight patients with PPA. The presence of the MMN suggests that the deviant stimulus [gǝ] was discriminated against the standard stimulus [bǝ] in these patients. No differences were found between the MMN components in the patients with the SV and the HC group. These findings indicate intact phoneme discrimination processes in patients with SV. However, differences in these phoneme discrimination processes were found between the HC group and the patients with the NFV, LV, and PPA-NOS based on the comparison of the mean amplitudes and onset latencies. The MMN was absent in case 3 with late-stage NFV and case 4 with the early-stage PPA-NOS. These results suggest that the two phonemes were not discriminated in these patients. In the remaining patients with the NFV and LV in which the MMN was present, the onset latency was delayed in both patients with the NFV and one patient with late-stage LV. In these patients, it might be possible that the access to the phoneme trace was delayed due to early perceptual deficits before the MMN response was initiated. Previous studies have already suggested the presence of early perceptual deficits in patients with NFV. Deficits in the discrimination of complex sound properties (Goll et al., 2010), the rhythm of tone sequences (Grube et al., 2016), pitch detection perception (Goll et al., 2011), and timbre perception (Goll et al., 2011) have been reported in patients with the NFV. Furthermore, there is evidence for peripheral hearing deficits in the NFV (Hardy et al., 2019). In contrast to this finding, the pure-tone audiometry and tympanometry performed in our study showed a sensorineural age-related hearing impairment in each patient. The number of included patients was, however, limited in our study and we did not measure the peripheral hearing function of our HC group. In future research, it might be important to further examine the early perceptual deficits and their influence on the consecutive phoneme perception and language comprehension processes.

The precise neural mechanisms underlying the MMN remain unclear but one of the theories that have been used to explain the manifestation of the MMN is the predictive coding theory (Chennu et al., 2013; Fitzgerald and Todd, 2020; Fong et al., 2020). According to this theory, the MMN reflects the level of prediction error when an unexpected stimulus is presented. Consequently, the delayed MMN in the patients with the NFV might reflect a delay in the bottom-up processes which pass forward the prediction error signals. A delayed reconciliation of predictions was also found in patients with the NFV in the study of Cope et al. (2017). Interestingly, the mean amplitude was increased at the frontal electrode sites in the two patients with early-stage NFV. In the context of the predictive coding theory, the increased amplitude might suggest an increased prediction error signal in comparison to the prediction error signal of the HC group. In conclusion, the MMN might help differentiate patients with the NFV and LV from HCs, and patients with the SV from patients with the NFV and the LV. In patients with the NFV and LV, deficits in phoneme discrimination processes were found but further research in larger patient groups is required to investigate the applicability of the MMN in the differential diagnosis and to explore the nature of these phoneme perception deficits. Our findings do not support the results of the study of Johnson et al. (2020) in which was suggested phoneme discrimination may help differentiate the LV from the NFV. An explanation for this might be that in our study two auditorily presented phonemes were used and in the behavioral task of the study of Johnson et al. (2020), minimal word pairs were administered. In the latter, the participants had to underline one of two written words that matched the spoken monosyllabic word.

The **P300** component was absent in four of the six patients with the NFV, SV, and PPA-NOS. More particularly, this absent P300 was found in two patients with the NFV (early- and late-stage), one patient with the SV (late-stage), and the patient with PPA-NOS. Although the P300 was present in one patient with the early-stage NFV and possibly present in one patient with the early-stage SV, these results indicate that deficits in phoneme categorization processes might be present in patients with the NFV, SV, and PPA-NOS. Since the results are variable in these variants, further research is required in larger patient groups. Interestingly, the P300 was present with an abnormal P300 pattern in both patients with the LV. The onset latency of the P300 was delayed and the P300 was prolonged with increased mean amplitude in both patients with the LV in comparison to the HC group. This P300 pattern was not found in any of the other included patients. The latency of the P300 is thought to index the stimulus categorization time and the amplitude of the P300 is associated with resource allocation. Consequently, the time required to categorize the stimulus as standard or deviant might be delayed and prolonged in the patients with the LV in comparison to the HC group. The increased mean amplitude and prolonged P300 in the patients with the LV might suggest that these patients devoted more effort to the task than the HC group (Luck and Kappenman, 2012). In conclusion, the results of the P300 are variable in the different variants of PPA. However, this study suggests a specific P300 pattern in patients with the LV that might distinguish them from patients with the NFV, the SV, and PPA-NOS. The nature of these deficits in the clinical variants of PPA still needs to be clarified in further research since the results in this study were based on a small patient group.

The most important limitation of this study was the number of patients with PPA included in this study. However, early- and late-stage patients with the three clinical variants of PPA were included. Further research in independent and larger patient groups of the various clinical variants is required. Due to the small patient group, the results were described on a single-subject level and compared to an age-matched HC group. The applied method might contribute to the diagnostic process in clinical practice and reveal information about the nature of the speech perception deficits in PPA. To our knowledge, this is the first study to apply *Z*-scores to compare the amplitudes and latencies of a single subject with an HC group. We used 0.1 as the threshold for alpha since this value is frequently used in behavioral linguistic and neuropsychological assessments, but further research should define which values are clinically significant. Furthermore, deficits in nonverbal sound processing have been reported as well in patients with PPA (Bozeat et al., 2000; Goll et al., 2010, 2011; Rohrer et al., 2012; Golden et al., 2015; Grube et al., 2016; Hardy et al., 2017a). Thus, the question may arise whether these results reflect deficits in the spectro-temporal analysis of auditory signals, the language-specific processing of phonemes, or a combination of both spectro-temporal and phoneme analysis (Becker and Reinvang, 2007). It might be interesting for future research to investigate the MMN and P300 components by oddball paradigms using various nonverbal and verbal stimuli such as tones, phonemes, and syllables. In addition, this study only examined one phonemic contrast (articulation place) and in patients with aphasia differences in the MMN and P300 amplitudes were found depending on the phonemic contrasts (Aerts et al., 2015). This might also be an interesting research area in further research. Finally, the P300 in particular is associated with attention and working memory abilities. These cognitive domains might also be affected in patients with PPA, particularly in the later disease stages. Further studies, which take these variables into account, will need to be undertaken.

The results of this study showed that the MMN might be valuable to differentiate the SV from the NFV and the LV and the P300 to differentiate the LV from the NFV and the SV, in the early as well as in the later disease stages. Since our results are based on a small patient group, further research in larger and independent patient groups is required to investigate the applicability of these components in the diagnostic process and to determine the nature of these speech perception deficits in the clinical variants of PPA. It might also be interesting to follow up on these components to investigate the decline of the phoneme perception processes in each variant and to measure neuroplasticity changes in PPA. In patients with post-stroke aphasia, previous research suggested that the MMN and P300 components might be considered as an indicator of auditory discrimination and categorization recovery (Ilvonen et al., 2003; Nolfe et al., 2006; Cocquyt et al., 2020). Additionally, individualized therapeutic interventions focused on improving or maintaining the phoneme perception abilities could be investigated in future research. These interventions might be determined by examining the MMN and P300 components in combination with a careful language and speech examination by the speech-language pathologist.

## Data Availability Statement

The original contributions presented in the study are included in the article/Supplementary Material, further inquiries can be directed to the corresponding author.

## Ethics Statement

The studies involving human participants were reviewed and approved by Ethics Committee of the University Hospital Ghent. The patients/participants provided their written informed consent to participate in this study. Written informed consent was obtained from the individual(s) for the publication of any potentially identifiable images or data included in this article.

## Author Contributions

JS and MDL contributed to the design of the experiments. TL and AS recruited the patients with PPA. JS contributed to the data collection, data analysis, interpretation of the results, and writing of the manuscript under the supervision of MDL and PM. MDL, PM, TL, MM, and AS provided critical feedback and helped shape the manuscript. All authors contributed to the article and approved the submitted version.

## Conflict of Interest

The authors declare that the research was conducted in the absence of any commercial or financial relationships that could be construed as a potential conflict of interest.
