# Supplementary material for: The Electrophysiological Correlates of Phoneme Perception in Primary Progressive Aphasia: A Preliminary Case Series
Source: Front Hum Neurosci. 2021 Jun 2;15:618549. doi: 10.3389/fnhum.2021.618549 (PMC8206281; doi:10.3389/fnhum.2021.618549)
Supplement: Supplementary file 1 [file Table_1.DOCX]

Supplementary Material

## MMN

Table 1. Raw values of the mean amplitudes of the difference waves (deviant – standard condition) for each included patient with PPA over the time windows 150ms – 250ms, 250ms – 350ms, and 350ms – 450ms at the frontal, central, parietal, left, midline, and right electrode sites.

| Case |  | 1 | 2 | 3 | 4 | 5 | 6 | 7 | 8 |
| --- | --- | --- | --- | --- | --- | --- | --- | --- | --- |
| Variant |  | Early NFV | Early NFV | Late NFV | Early mixed | Early LV | Late LV | Early SV | Late SV |
| 150-250 | F | -2.45 | -0.62 | -0.63 | -0.03 | -1.17 | -0.25 | -1.61 | -1.88 |
|  | C | -1.25 | -0.65 | -0.62 | -0.25 | -1.17 | -0.43 | -1.51 | -1.16 |
|  | P | 0.02 | -0.59 | -0.81 | -0.79 | -1 | -0.23 | -1.2 | -0.29 |
|  | L | -1.52 | -0.68 | -0.66 | -0.52 | -0.88 | -0.3 | -1.55 | -1.32 |
|  | M | -1.31 | -0.59 | -0.81 | -0.31 | -1.18 | -0.35 | -1.45 | -1.19 |
|  | R | -0.86 | -0.59 | -0.6 | -0.24 | -1.29 | -0.26 | -1.32 | -0.81 |
| 250-350 | F | -2.95 | -2.35 | -0.37 | -0.48 | -0.69 | -0.03 | -0.73 | -1.29 |
|  | C | -1.22 | -1.7 | -0.52 | -0.53 | -0.86 | 0.09 | -0.78 | -0.58 |
|  | P | 0.33 | -0.88 | -0.69 | -0.67 | -1.14 | 0.35 | -0.88 | 0.1 |
|  | L | -1.3 | -1.67 | -0.47 | -0.65 | -0.5 | 0.24 | -0.71 | -0.66 |
|  | M | -1.43 | -1.59 | -0.71 | -0.6 | -0.92 | 0.13 | -0.81 | -0.63 |
|  | R | -1.11 | -1.68 | -0.41 | -0.44 | -1.27 | 0.04 | -0.87 | -0.47 |
| 350-450 | F | -2.3 | 0.91 | -0.33 | -0.28 | -0.62 | -0.73 | -0.41 | -1.14 |
|  | C | -0.98 | 0.14 | -0.58 | -0.49 | -0.71 | -0.04 | -0.54 | -0.5 |
|  | P | 0.25 | -0.28 | -0.6 | -0.66 | -0.81 | 0.8 | -0.88 | -0.07 |
|  | L | -1.05 | 0.27 | -0.39 | -0.29 | -0.68 | 0.04 | -0.75 | -0.46 |
|  | M | -1.16 | 0.42 | -0.69 | -0.51 | -0.74 | 0.05 | -0.55 | -0.72 |
|  | R | -0.83 | 0.09 | -0.44 | -0.64 | -0.72 | -0.07 | -0.53 | -0.54 |

Abbreviations: F = frontal, C = central, P = parietal, L = left, M = midline, R = right, NFV = nonfluent variant, LV = logopenic variant, SV = semantic variant.

|  | Decreased mean amplitude |
| --- | --- |
|  | Increased mean amplitude |

Table 2. Raw values of the onset latencies of the difference waves (deviant – standard condition) for each included patient with PPA at the frontal, central, parietal, left, midline, and right electrode sites.

| Case | 1 | 2 | 3 | 4 | 5 | 6 | 7 | 8 |
| --- | --- | --- | --- | --- | --- | --- | --- | --- |
| Variant | Early NFV | Early NFV | Late NFV | Early mixed | Early LV | Late LV | Early SV | Late SV |
| F | 236 | 239.33 | 210.67 | 265.33 | 219.33 | 212 | 193.33 | 199.33 |
| C | 226 | 226 | 220.67 | 222.67 | 222.67 | 173.33 | 198.67 | 190 |
| P | 188 | 230 | 218.67 | 212 | 230 | 172 | 215.33 | 181.33 |
| L | 206.67 | 237.33 | 215.33 | 216.67 | 221.33 | 173.33 | 195.33 | 190.67 |
| M | 233.33 | 228.67 | 221.33 | 242 | 224 | 176.67 | 202 | 194 |
| R | 210 | 229.33 | 213.33 | 241.33 | 226.67 | 207.33 | 210 | 186 |

Abbreviations: F = frontal, C = central, P = parietal, L = left, M = midline, R = right, NFV = nonfluent variant, LV = logopenic variant, SV = semantic variant.

|  | Delayed onset latency |
| --- | --- |

Table 3. Z-scores of the mean amplitudes of the difference waves (deviant – standard condition) for each included patient with PPA over the time windows 150ms – 250ms, 250ms – 350ms, and 350ms – 450ms at the frontal, central, parietal, left, midline, and right electrode sites.

| Case |  | 1 | 2 | 3 | 4 | 5 | 6 | 7 | 8 |
| --- | --- | --- | --- | --- | --- | --- | --- | --- | --- |
| Variant |  | Early NFV | Early NFV | Late NFV | Early mixed | Early LV | Late LV | Early SV | Late SV |
| 150-250 | F | -1.12 | 1.29 | 1.28 | 2.06 | 0.57 | 1.78 | -0.02 | -0.37 |
|  | C | 0.12 | 0.94 | 0.97 | 1.48 | 0.23 | 1.23 | -0.23 | 0.25 |
|  | P | 1.33 | 0.31 | -0.06 | -0.01 | -0.37 | 0.91 | -0.69 | 0.82 |
|  | L | -0.58 | 0.9 | 0.94 | 1.17 | 0.55 | 1.56 | -0.62 | -0.23 |
|  | M | 0.03 | 1.03 | 0.72 | 1.42 | 0.21 | 1.36 | -0.16 | 0.19 |
|  | R | 0.5 | 0.88 | 0.87 | 1.39 | -0.13 | 1.36 | -0.18 | 0.56 |
| 250-350 | F | -2.37 | -1.63 | 0.82 | 0.68 | 0.42 | 1.23 | 0.37 | -0.32 |
|  | C | -0.46 | -1.07 | 0.41 | 0.4 | -0.01 | 1.18 | 0.09 | 0.34 |
|  | P | 1.24 | -0.59 | -0.31 | -0.28 | -0.99 | 1.27 | -0.6 | 0.9 |
|  | L | -0.83 | -1.42 | 0.48 | 0.19 | 0.43 | 1.61 | 0.09 | 0.18 |
|  | M | -0.77 | -0.98 | 0.14 | 0.27 | -0.12 | 1.2 | 0.01 | 0.24 |
|  | R | -0.44 | -1.22 | 0.52 | 0.48 | -0.66 | 1.13 | -0.11 | 0.44 |
| 350-450 | F | -2.41 | 1.5 | -0.02 | 0.05 | -0.37 | -0.5 | -0.1 | -1 |
|  | C | -0.84 | 0.54 | -0.35 | -0.23 | -0.5 | 0.32 | -0.3 | -0.25 |
|  | P | 0.88 | 0.14 | -0.32 | -0.42 | -0.63 | 1.68 | -0.73 | 0.42 |
|  | L | -1.14 | 0.81 | -0.17 | -0.02 | -0.59 | 0.48 | -0.71 | -0.27 |
|  | M | -1.01 | 0.97 | -0.42 | -0.19 | -0.49 | 0.5 | -0.24 | -0.46 |
|  | R | -0.62 | 0.59 | -0.1 | -0.37 | -0.49 | 0.39 | -0.22 | -0.24 |

Abbreviations: F = frontal, C = central, P = parietal, L = left, M = midline, R = right, NFV = nonfluent variant, LV = logopenic variant, SV = semantic variant.

|  | Decreased mean amplitude |
| --- | --- |
|  | Increased mean amplitude |

Table 4. Z-scores of the onset latencies of the difference waves (deviant – standard condition) for each included patient with PPA at the frontal, central, parietal, left, midline, and right electrode sites.

| Case | 1 | 2 | 3 | 4 | 5 | 6 | 7 | 8 |
| --- | --- | --- | --- | --- | --- | --- | --- | --- |
| Variant | Early NFV | Early NFV | Late NFV | Early mixed | Early LV | Late LV | Early SV | Late SV |
| F | 1.66 | 1.86 | 0.12 | 3.44 | 0.65 | 0.2 | -0.93 | -0.57 |
| C | 0.91 | 0.91 | 0.58 | 0.7 | 0.7 | -2.37 | -0.79 | -1.33 |
| P | -1.35 | 0.65 | 0.11 | -0.21 | 0.65 | -2.12 | -0.05 | -1.67 |
| L | -0.37 | 1.46 | 0.15 | 0.23 | 0.51 | -2.35 | -1.04 | -1.32 |
| M | 1.21 | 0.95 | 0.54 | 1.7 | 0.69 | -1.97 | -0.55 | -0.99 |
| R | -0.13 | 1.09 | 0.08 | 1.84 | 0.92 | -0.29 | -0.13 | -1.63 |

Abbreviations: F = frontal, C = central, P = parietal, L = left, M = midline, R = right, NFV = nonfluent variant, LV = logopenic variant, SV = semantic variant.

|  | Delayed onset latency |
| --- | --- |

## P300

Table 5. Raw values of the mean amplitudes of the difference waves (deviant – standard condition) for each included patient with PPA over the time windows 350ms – 550ms, 550ms – 750ms, and 750ms – 950ms at the frontal, central, parietal, left, midline, and right electrode sites.

| Case |  | 1 | 2 | 3 | 4 | 5 | 6 | 7 | 8 |
| --- | --- | --- | --- | --- | --- | --- | --- | --- | --- |
| Variant |  | Early NFV | Early NFV | Late NFV | Early mixed | Early LV | Late LV | Early SV | Late SV |
| 350-550 | F | -2.97 | 2.21 | -1.28 | -0.74 | -1.34 | -0.16 | 0.43 | -1.3 |
|  | C | -2.75 | 2.36 | -0.97 | -0.68 | -1.29 | 1.9 | 0.18 | -1.36 |
|  | P | -1.58 | 3.92 | -0.58 | -1.42 | -0.51 | 3.65 | 1.06 | -0.93 |
|  | L | -2.27 | 3.28 | -0.67 | -1.08 | -1.30 | 1.77 | -0.34 | -0.8 |
|  | M | -2.72 | 3.41 | -0.99 | -1.08 | -1.20 | 2.05 | 0.48 | -1.75 |
|  | R | -2.32 | 1.8 | -1.17 | -0.68 | -0.63 | 1.58 | 1.53 | -1.04 |
| 550-750 | F | -2.99 | -2.15 | -0.97 | -1.47 | 1.21 | 0.77 | 0.35 | 0.14 |
|  | C | -2.72 | -1.94 | -0.76 | -1.48 | 1.79 | 2.82 | 0.68 | -0.88 |
|  | P | -0.49 | 1.43 | -0.42 | -2.23 | 1.88 | 3.8 | 2.88 | -0.5 |
|  | L | -2.3 | -0.55 | -0.43 | -1.77 | 1.49 | 1.62 | 1.42 | -0.22 |
|  | M | -2.12 | -1.17 | -0.74 | -2.09 | 1.80 | 2.99 | 0.99 | -1.14 |
|  | R | -1.79 | -0.94 | -0.98 | -1.32 | 1.59 | 2.78 | 1.5 | 0.12 |
| 750-950 | F | -4.52 | -1.29 | -0.82 | -1.84 | 0.89 | 0.83 | -0.53 | 0.03 |
|  | C | -2.71 | -1.11 | -0.32 | -1.48 | 1.96 | 1.76 | 0.07 | -1.28 |
|  | P | 1.33 | -0.41 | -0.03 | -1.35 | 2.23 | 2.97 | 1.92 | -0.74 |
|  | L | -2.74 | -1.2 | 0.18 | -1.69 | 1.16 | 1.62 | 1.01 | -0.49 |
|  | M | -1.43 | -1.75 | -0.28 | -1.62 | 1.90 | 2.46 | -0.25 | -2.06 |
|  | R | -1.74 | 0.13 | -1.06 | -1.37 | 2.02 | 1.48 | 0.7 | 0.56 |

Abbreviations: F = frontal, C = central, P = parietal, L = left, M = midline, R = right, NFV = nonfluent variant, LV = logopenic variant, SV = semantic variant.

|  | Decreased mean amplitude |
| --- | --- |
|  | Increased mean amplitude |

Table 6. Raw values of the onset latencies of the difference waves (deviant – standard condition) for each included patient with PPA at the frontal, central, parietal, left, midline, and right electrode sites.

| Case | 1 | 2 | 3 | 4 | 5 | 6 | 7 | 8 |
| --- | --- | --- | --- | --- | --- | --- | --- | --- |
| Variant | Early NFV | Early NFV | Late NFV | Early mixed | Early LV | Late LV | Early SV | Late SV |
| F | 352 | 426.67 | 626 | 422 | 637.33 | 649.33 | 384.67 | 699.33 |
| C | 352 | 442 | 605.33 | 414.67 | 662.67 | 514 | 510.67 | 467.33 |
| P | 820 | 464 | 402.67 | 432.67 | 668.00 | 502.67 | 553.33 | 550 |
| L | 498.67 | 427.33 | 620.67 | 402 | 638.67 | 554 | 546 | 487.33 |
| M | 519.33 | 432 | 644.67 | 412.67 | 659.33 | 551.33 | 436.67 | 549.33 |
| R | 506 | 473.33 | 368.67 | 454.67 | 670.00 | 560.67 | 466 | 680 |

Abbreviations: F = frontal, C = central, P = parietal, L = left, M = midline, R = right, NFV = nonfluent variant, LV = logopenic variant, SV = semantic variant.

|  | Delayed onset latency |
| --- | --- |

Table 7. Z-scores of the mean amplitudes of the difference waves (deviant – standard condition) for each included patient with PPA over the time windows 350ms – 550ms, 550ms – 750ms, and 750ms – 950ms at the frontal, central, parietal, left, midline, and right electrode sites.

| Case |  | 1 | 2 | 3 | 4 | 5 | 6 | 7 | 8 |
| --- | --- | --- | --- | --- | --- | --- | --- | --- | --- |
| Variant |  | Early NFV | Early NFV | Late NFV | Early mixed | Early LV | Late LV | Early SV | Late SV |
| 350-550 | F | -1.42 | 0.31 | -0.85 | -0.67 | -0.88 | -0.48 | -0.28 | -0.86 |
|  | C | -1.17 | 0.3 | -0.66 | -0.58 | -0.75 | 0.17 | -0.33 | -0.77 |
|  | P | -1.25 | 0.18 | -1 | -1.21 | -0.98 | 0.11 | -0.57 | -1.09 |
|  | L | -1.3 | 0.59 | -0.76 | -0.9 | -0.97 | 0.07 | -0.64 | -0.8 |
|  | M | -1.4 | 0.38 | -0.9 | -0.92 | -0.96 | -0.02 | -0.47 | -1.12 |
|  | R | -1.47 | -0.12 | -1.09 | -0.93 | -0.92 | -0.19 | -0.21 | -1.05 |
| 550-750 | F | -0.8 | -0.44 | 0.06 | -0.15 | 0.98 | 0.8 | 0.62 | 0.53 |
|  | C | -1.13 | -0.85 | -0.42 | -0.68 | 0.52 | 0.9 | 0.11 | -0.46 |
|  | P | -1.1 | -0.44 | -1.08 | -1.7 | -0.29 | 0.37 | 0.05 | -1.11 |
|  | L | -1.25 | -0.44 | -0.39 | -1.01 | 0.51 | 0.56 | 0.47 | -0.29 |
|  | M | -1.11 | -0.73 | -0.56 | -1.1 | 0.46 | 0.94 | 0.14 | -0.72 |
|  | R | -1.28 | -0.88 | -0.9 | -1.06 | 0.32 | 0.88 | 0.27 | -0.38 |
| 750-950 | F | -1.46 | 0 | 0.21 | -0.25 | 0.99 | 0.96 | 0.34 | 0.6 |
|  | C | -0.92 | -0.14 | 0.24 | -0.32 | 1.36 | 1.26 | 0.44 | -0.22 |
|  | P | 0.56 | -0.22 | -0.04 | -0.64 | 0.97 | 1.3 | 0.83 | -0.36 |
|  | L | -0.93 | -0.05 | 0.75 | -0.33 | 1.31 | 1.58 | 1.22 | 0.36 |
|  | M | -0.29 | -0.44 | 0.23 | -0.38 | 1.23 | 1.49 | 0.25 | -0.58 |
|  | R | -0.99 | 0.16 | -0.57 | -0.76 | 1.32 | 0.99 | 0.51 | 0.43 |

Abbreviations: F = frontal, C = central, P = parietal, L = left, M = midline, R = right, NFV = nonfluent variant, LV = logopenic variant, SV = semantic variant.

|  | Decreased mean amplitude |
| --- | --- |
|  | Increased mean amplitude |

Table 8. Z-scores of the onset latencies of the difference waves (deviant – standard condition) for each included patient with PPA at the frontal, central, parietal, left, midline, and right electrode sites.

| Case | 1 | 2 | 3 | 4 | 5 | 6 | 7 | 8 |
| --- | --- | --- | --- | --- | --- | --- | --- | --- |
| Variant | Early NFV | Early NFV | Late NFV | Early mixed | Early LV | Late LV | Early SV | Late SV |
| F | -1.15 | -0.53 | 1.11 | -0.57 | 1.20 | 1.3 | -0.88 | 1.71 |
| C | -1.72 | -0.43 | 1.91 | -0.83 | 2.73 | 0.6 | 0.55 | -0.07 |
| P | 4.03 | -0.38 | -1.14 | -0.76 | 2.15 | 0.1 | 0.73 | 0.69 |
| L | 0.13 | -0.7 | 1.56 | -1 | 1.77 | 0.78 | 0.68 | 0 |
| M | 0.34 | -0.65 | 1.75 | -0.87 | 1.91 | 0.7 | -0.6 | 0.67 |
| R | 0.39 | -0.12 | -1.78 | -0.42 | 2.99 | 1.26 | -0.24 | 3.14 |

Abbreviations: F = frontal, C = central, P = parietal, L = left, M = midline, R = right, NFV = nonfluent variant, LV = logopenic variant, SV = semantic variant.

|  | Delayed onset latency |
| --- | --- |
